# How Well Are Dental Students Equipped to Handle Traumatic Dental Injuries in Children?

**DOI:** 10.1111/aej.70074

**Published:** 2026-04-06

**Authors:** Sobia Zafar, Luan Lin Jovita Cheong, Vonn Ler, Anastasia Min Teh, Paul Abbott

**Affiliations:** ^1^ School of Dentistry The University of Queensland Australia; ^2^ School of Dentistry The University of Western Australia Australia

**Keywords:** confidence, dental, knowledge, paediatric, trauma

## Abstract

To evaluate dental students' knowledge and confidence in managing dental trauma (TDI). An observational questionnaire‐based study involving fourth‐year BDSc (Hons) students from 2022 and 2023 at UQ, following their Traumatic Dental Injury module. A 44‐item questionnaire assessed knowledge in managing TDI in primary dentition and fractures, subluxations and avulsion of permanent teeth. Confidence was self‐reported. Scores were categorised as Below average (0%–49.9%), Average (50%–74.9%) and Above average (75%–100%). Data analysis used Jamovi (Version 2.3.18), with Prism GraphPad (Version 10.0.2) for graphs. Of 151 students, 118 completed the questionnaire (78.1% response rate). Average knowledge was 59.6%, with better understanding of primary dentition management (76.5%) and poor knowledge of avulsion management (42.6%). Most students (70.3%) demonstrated average knowledge, but 83.9% reported low confidence. Students had average knowledge but low confidence in managing TDI cases, highlighting the need for more undergraduate and postgraduate clinical exposure and preclinical simulations.

## 1 Introduction

Traumatic dental injuries (TDIs) occur when there is a direct impact or force applied to a tooth and/or its surrounding soft and/or hard tissues [1]. The global TDI incidence rate stands at 2.82 per 100 persons per year, with a prevalence of 15.2% in permanent teeth and 22.7% in primary teeth [2]. Approximately one in every three preschool children has experienced trauma to their primary teeth while one in every four school children has experienced trauma to their permanent dentition [3]. TDIs occur primarily in children and are associated with their poor motor skills, their participation in sports and their involvement in physical fights [4]. TDIs in children include luxations, crown fractures, crown‐root fractures, root fractures, alveolar bone fractures and avulsion [3, 4]. TDIs cause acute pain and may have enduring consequences, particularly when injuries involve immature permanent teeth or primary teeth adjacent to developing successors. These sequelae can extend into adolescence and adulthood, adversely affecting aesthetics, psychosocial development, masticatory function and overall quality of life.

Prompt initial management and subsequent maintenance of a traumatised tooth are critical determinants of its recovery and longevity [2, 4]. Unfortunately, appropriate and immediate treatment is often not administered due to inadequate knowledge of managing TDIs. The most serious consequences of inadequate treatment include tooth discolouration, pulp necrosis, infection of the root canal system, tooth resorption, ankylosis, loss of gingival attachment and subsequent tooth loss [5]. The effects of untreated or poorly treated TDIs go beyond above consequences, adversely affecting key domains of child development such as functional capacity, social interactions and psychological well‐being, and thereby compromising overall oral health‐related quality of life (OHRQoL) of these children [6, 7, 8].

The International Association for Dental Traumatology (IADT) guidelines are regarded as the gold standards for the management of dental trauma by various dental societies worldwide [9, 10, 11, 12]. As an internationally recognised set of guidelines, they have been taught in dental schools and serve to educate dental students about the standardised professional approach for managing TDIs [9, 10, 11, 12]. It is imperative that dental students, who will graduate to become dental practitioners, are competent in handling TDIs in both the primary and permanent dentitions as they are often the first point of contact to provide immediate treatment [4, 13].

The importance of possessing adequate knowledge in TDI management has been highlighted in several research studies globally that have assessed the knowledge level of TDI management in dental practitioners, dental students, as well as school teachers, training coaches and parents [1, 4, 9, 10, 11, 12, 13]. However, such studies are lacking locally, with no reported studies that have assessed the knowledge of TDI management in Australian dental students. Research studies conducted on Japanese and Saudi Arabian dental students revealed that less than 50% of the students were able to correctly identify the appropriate treatment protocols regarding TDI management [13, 14]. International studies largely find students underprepared for TDI management [13, 15, 16, 17], however, an Australian study reporting satisfactory graduate knowledge suggests Australian student results may differ [1]. Hence, further investigations measuring the knowledge of TDI management need to be undertaken on Australian dental students to better understand this relationship.

The myriad of research articles have utilised different surveys to measure TDI management knowledge in their corresponding dental schools, but they did not base their surveys on the IADT guidelines [1, 2, 13, 14]. This has resulted in a lack of test standardisation, inadvertently causing direct comparisons of the TDI knowledge level of dental students from different countries to be difficult. In addition, many of their surveys involve only a small number of questions to test TDI knowledge, resulting in limited data being obtained [2, 13, 14]. A more comprehensive questionnaire based on the different sections of the IADT guidelines would be more accurate in measuring the TDI knowledge of dental students. Furthermore, there are few studies comparing the association between knowledge and confidence in TDI management. It is crucial to assess the existing knowledge of dental students regarding TDI management so that inadequacies in the dental curriculum can be addressed and further improved [1, 2, 13, 18]. Therefore, the aim of this study was to assess the knowledge and confidence levels of dental students regarding TDI management of primary and young permanent teeth in children in an Australian school.

## 2 Materials and Methods

This study was a quantitative cross‐sectional study that measured the knowledge and confidence levels of dental students regarding TDI management across two cohorts (2022 and 2023) of fourth‐year dental students at The University of Queensland (UQ) via a questionnaire. This study was approved by the Human Research Ethics Committee (Approval No 2020/HE000880).

### 2.1 Study Participants

The study involved the completion of a questionnaire by dental students in their penultimate (fourth) year of the degree, and who were enrolled in the Bachelor of Dental Science (Honours) degree at UQ in the academic years of 2022 and 2023. All fourth‐year dental students enrolled in DENT4071 in Semester 1 of each of these years were invited to participate. The TDI module, taught in the first semester of the fourth‐year curriculum, consisted of 9 h of lectures regarding TDI management and 3 h of preclinical sessions with simulations that allow students to practise the splinting of an avulsed tooth. The questionnaire, information sheet and its consent form were distributed physically to the dental students during their curriculum. Data collection was carried out from April 2022 to April 2023. Participation in this study was voluntary. Questionnaires were collected separately from the consent form to ensure anonymity and confidentiality.

### 2.2 Study Tool

A questionnaire comprising 44 questions was designed for this study. The questions were adapted from published studies based on the IADT guidelines and modified to ensure they comprehensively covered knowledge and confidence in trauma management in both the primary and permanent dentitions [1, 9, 10, 11, 12]. The questionnaire was piloted by administering it to 10 specialists endodontic (*n* = 5) and paediatric dentists (*n* = 5) who were asked to provide comments. The questionnaire was then modified according to the feedback received.

The students undertook the questionnaire after completion of the TDI module. The questionnaire consisted of five sections corresponding to the sections of the IADT guidelines [9, 10, 11, 12]. *Section A* recorded participant demographics. *Section B* assessed knowledge of TDI management in the primary dentition. *Section C* assessed knowledge of managing fractures and subluxation in the permanent dentition. *Section D* assessed knowledge of managing avulsion of permanent teeth. *Section E* was a self‐assessment of participants' confidence and perception in handling TDI cases.

### 2.3 TDI Knowledge Level

The knowledge level of TDI management in dental students was obtained by tabulating a score from Sections B, C and D of the questionnaire. A knowledge score out of 44 points was obtained for each student where a correct answer was assigned one point and an incorrect answer was assigned a zero score. The knowledge scores collected were converted to a percentage score and categorised into three knowledge levels: Below average (0%–49.9%), Average (50%–74.9%) and Above average (75%–100%).

### 2.4 Confidence Level in Handling TDI

This information was obtained from Section E of the questionnaire. Questions related to confidence level in TDI management were measured on a Likert scale, with Strongly Disagree assigned a zero score to Strongly Agree being assigned four points, with a maximum possible overall score of 20 points. Confidence scores were converted to percentages and categorised into three confidence levels: Below average (0%–49.9%), Average (50%–74.9%) and Above average (75%–100%).

### 2.5 Statistical Analysis

The data were tabulated in Microsoft Excel for MacOS Version 16.66.1 (Microsoft, USA) and imported to Jamovi for MacOS, Version 2.3.18 (phpBB Group, Australia). The data were manipulated and categorised into the aforementioned categories. Appropriate graphs were constructed using GraphPad Prism for MacOS, Version 10.0.2 (GraphPad Software, San Diego, CA, USA). Assuming that all dental students have access to the materials and methods used in the dental trauma module, the scores tabulated should accurately reflect their knowledge levels. The results collected were presented as demographics, stating the count and their percentages in table format. Independent *t*‐tests were used to compare the results of dichotomised variables including gender and clinical experience against knowledge levels. One‐way ANOVA was used to compare results with more than two responses including: age, received dental trauma course in school, heard of IADT, read IADT and confidence levels against knowledge levels. The statistical significance was set at *p* < 0.05.

## 3 Results

### 3.1 Demographics

There were 151 students enrolled in the two cohorts (81 in 2022; 70 in 2023). A total of 118 students completed the survey (overall response rate 78.1%)—61 were fourth year dental students in 2022 and 57 fourth year dental students in 2023. The response rates for each cohort were 75.3% in 2022 and 81.4% in 2023. The distribution of demographics and their mean scores are summarised in Table 1. There was no statistical difference between knowledge scores and the demographics, except for those who had heard of the IADT guidelines (*p* = 0.003) and those who read the IADT guidelines (*p* = 0.003). There was no statistical difference between confidence scores and the demographics.

**TABLE 1:** Mean confidence and knowledge scores of demographics.

Demographics	Participants *N* (%)	Knowledge score		Confidence score	
		Total Mean ± SD	*p*	Total Mean ± SD	*p*
Age					
< 20	4 (3.39)	28.8 ± 5.0	0.679	8.5 ± 6.9	0.912
20–24	111 (94.1)	26.2 ± 5.8		6.9 ± 4.3	
25–29	3 (2.54)	25.7 ± 10.4		7.0 ± 2.7	
30–34	0 (0.0)	0.0		0.0	
> 35	0 (0.0)	0.0		0.0	
Sex					
Male	50 (42.4)	25.8 ± 6.9	0.456	7.7 ± 4.6	0.106
Female	68 (57.6)	26.6 ± 4.9		6.4 ± 4.0	
Received dental trauma module in school					
No	3 (2.5)	18.3 ± 9.9	—	4.3 ± 4.0	—
Yes	114 (96.6)	26.4 ± 5.6		7.0 ± 4.3	
Unsure	1 (0.8)	29.0		3.0	
Clinical experience with dental trauma in children					
No	110 (93.2)	26.3 ± 5.8	0.684	6.7 ± 4.0	0.142
Yes	8 (6.8)	25.4 ± 5.9		10.5 ± 6.5	
Heard of IADT					
No	14 (11.9)	18.2 ± 6.0	0.003	6.4 ± 3.7	0.875
Yes	99 (83.9)	27.6 ± 4.8		7.0 ± 4.5	
Unsure	4 (3.4)	21.3 ± 8.0		7.0 ± 2.8	
No answer	1 (0.8)	—		—	
Read IADT					
No	41 (34.7)	26.2 ± 6.0	0.003	6.2 ± 3.7	0.148
Yes	46 (39.0)	28.1 ± 4.6		8.0 ± 5.1	
Unsure	16 (13.6)	23.5 ± 6.2		6.4 ± 3.5	
No answer	15 (12.7)	—		—	

### 3.2 Assessment of Knowledge in Different Areas of TDI Management

Knowledge of managing avulsion in permanent teeth was the poorest section (42.6% correct answers), followed by fractures and subluxation in permanent teeth (60.9% correct answers), while trauma management in primary teeth was the best section (76.5% correct answers). The distribution of correct answers for single‐answer questions in Sections B, C and D is shown in Table 2, and the distribution of responses for multiple‐answer questions is summarised in Table 3.

**TABLE 2:** Distribution of correct answers for single‐response questions based on participants' current knowledge of trauma management in primary teeth, and fractures, subluxation and avulsions of permanent teeth.

Statements	Year 4 (2022) *n* (%)	Year 4 (2023) *n* (%)	Total *n* (%)
Should an avulsed primary tooth be replanted?			
Correct answer	42 (68.9)	39 (68.4)	81 (68.7)
Incorrect answer	19 (31.1)	18 (31.6)	37 (31.3)
Are pulp sensibility tests conducted in primary teeth immediately after trauma more reliable?			
Correct answer	55 (90.2)	42 (73.7)	97 (82.2)
Incorrect answer	6 (9.8)	15 (26.3)	21 (17.8)
What is the least important clinical parameter in the management of a traumatised maxillary primary central incisor?			
Correct answer	54 (88.5)	21 (36.8)	75 (63.6)
Incorrect answer	7 (11.5)	36 (63.2)	43 (36.4)
Which of the following injury would most likely result in a concomitant alveolar fracture?			
Correct answer	45 (73.8)	44 (77.2)	89 (75.4)
Incorrect answer	16 (26.2)	13 (22.8)	29 (24.6)
What is the appropriate splinting duration for a permanent central incisor that sustained a lateral luxation injury?			
Correct answer	33 (54.1)	35 (61.4)	68 (57.6)
Incorrect answer	28 (45.9)	22 (38.6)	50 (42.4)
What is the treatment required for a root‐fractured central incisor with a degree of mobility and is tender to percussion?			
Correct answer	26 (42.6)	22 (38.6)	48 (40.7)
Incorrect answer	35 (57.4)	34 (59.6)	69 (58.5)
No answer	0 (0.0)	1 (1.8)	1 (0.8)
What trauma is sustained by the tooth, with two periapical radiographs taken with different vertical angulations shown below?			
Correct answer	31 (50.8)	24 (42.1)	55 (46.6)
Incorrect answer	30 (49.2)	33 (57.9)	63 (53.4)
What type of resorption is evident in the central incisor which has a history of trauma more than 40 years ago?			
Correct answer	49 (80.3)	51 (89.5)	100 (84.7)
Incorrect answer	12 (19.7)	6 (10.5)	18 (15.3)
Which of the following injury sustained to a permanent tooth, should endodontic treatment always be commenced in?			
Correct answer	16 (26.2)	23 (40.4)	39 (33.1)
Incorrect answer	45 (73.8)	33 (57.9)	78 (66.1)
No answer	0 (0.0)	1 (1.8)	1 (0.8)
Is it advantageous to use corticosteroids for the following injury where endodontic treatment was commenced?			
Correct answer	38 (62.3)	30 (52.6)	68 (57.6)
Incorrect answer	23 (37.7)	26 (45.6)	49 (41.5)
No answer	0 (0.0)	1 (1.8)	1 (0.8)
Is endodontics indicated where the permanent central incisor has sustained a trauma and is unresponsive to pulp tests?			
Correct answer	53 (86.9)	38 (66.7)	91 (77.1)
Incorrect answer	8 (13.1)	19 (33.3)	27 (22.9)
Which of the following is the best option for the emergency management of an avulsed tooth?			
Correct answer	50 (82.0)	36 (63.2)	86 (72.9)
Incorrect answer	11 (18.0)	20 (35.1)	31 (26.3)
No Answer	0 (0.0)	1 (1.7)	1 (0.8)
What is the duration in which an avulsed tooth should be stored in milk in order to achieve reliable periodontal healing?			
Correct answer	18 (29.5)	34 (59.6)	52 (44.1)
Incorrect answer	43 (70.5)	23 (40.4)	66 (55.9)
Is the choice of treatment for an avulsed permanent tooth related to the maturity of the root (open or closed apex)?			
Correct answer	55 (90.1)	40 (70.2)	95 (80.5)
Incorrect answer	4 (6.6)	7 (12.3)	11 (9.3)
Unsure	2 (3.3)	10 (17.5)	12 (10.2)
What type of resorption is evident in the central incisors that were avulsed 1 week ago and replanted after 30 min?			
Correct answer	21 (34.4)	35 (61.4)	56 (47.5)
Incorrect answer	40 (65.6)	22 (38.6)	62 (52.5)
What type of resorption is evident in the avulsed incisor that was replanted 3 years earlier with no treatment undertaken?			
Correct answer	40 (65.6)	40 (70.2)	80 (67.8)
Incorrect answer	21 (34.4)	17 (29.8)	38 (32.2)
What is the most appropriate treatment following avulsion of a maxillary central incisor in which a wire splint was placed?			
Correct answer	14 (23.0)	8 (14.0)	22 (18.6)
Incorrect answer	47 (77.0)	49 (86.0)	96 (81.4)
What should the diameter of the wire required for a flexible splint be?			
Correct answer	46 (75.4)	20 (16.1)	66 (55.9)
Incorrect answer	15 (24.6)	37 (64.9)	52 (44.1)
When endodontic treatment is indicated, when should treatment be initiated following post‐replantation?			
Correct answer	11 (18.0)	4 (7.0)	15 (12.7)
Incorrect answer	49 (80.4)	47 (82.5)	96 (81.4)
Unsure	1 (1.6)	6 (10.5)	7 (5.9)
Following an avulsion injury, what is the duration of extra‐alveolar dry time in which most PDL cells become nonviable?			
Correct answer	14 (23.0)	8 (14.0)	22 (18.6)
Incorrect answer	45 (73.7)	46 (80.7)	91 (77.1)
Unsure	2 (3.3)	3 (5.3)	5 (4.3)
Following an avulsion, what is the most likely scenario where the tooth was placed in milk and replanted after 60 min?			
Correct answer	41 (67.2)	31 (54.4)	72 (61.0)
Incorrect answer	20 (32.8)	26 (45.6)	46 (39.0)

**TABLE 3:** Distribution of responses for multiple‐answer questions in primary teeth, fractures, subluxation and avulsions of permanent teeth.

Statements	Year 4 (2022)		Year 4 (2023)		Total	
	Chosen	Not chosen	Chosen	Not chosen	Chosen	Not chosen
**Tdi Injuries to Primary Teeth**						
How should parents care for their child's injured tooth in the immediate post‐trauma period and prevent further injury						
Clean affected area with soft brush or cotton swab*	49 (80.3)	12 (19.7)	34 (59.6)	23 (40.4)	83 (70.3)	35 (29.7)
Use alcohol‐free chlorhexidine gluconate 0.12% mouthrinse	53 (86.9)	8 (13.1)	47 (82.5)	10 (17.5)	100 (84.7)	18 (15.3)
Care should be taken when eating to avoid further trauma*	51 (83.6)	10 (16.4)	44 (77.2)	13 (22.8)	95 (80.5)	23 (19.5)
Unsure	5 (8.2)	56 (91.8)	6 (10.5)	51 (89.5)	11 (9.3)	107 (90.7)
What are some possible complications that may occur following dental trauma, which parents or caregivers should take note of						
Swelling*	60 (98.4)	1 (1.6)	52 (91.2)	5 (8.8)	112 (94.9)	6 (5.1)
Increased mobility*	58 (95.1)	3 (4.9)	54 (94.7)	3 (5.3)	112 (94.9)	6 (5.1)
Presence of sinus tract*	39 (63.9)	22 (36.1)	44 (77.2)	13 (22.8)	83 (70.3)	35 (29.7)
Pain*	60 (98.4)	1 (1.6)	52 (91.2)	5 (8.8)	112 (94.9)	6 (5.1)
Infection*	52 (85.2)	9 (14.8)	48 (84.2)	9 (15.8)	100 (84.7)	18 (15.3)
Apical radiolucency	39 (63.9)	22 (36.1)	28 (49.1)	29 (50.9)	67 (56.8)	51 (43.2)
Abscess	40 (65.6)	21 (34.4)	41 (71.9)	16 (28.1)	81 (68.6)	37 (31.4)
Unsure	1 (1.6)	60 (98.3)	1 (1.8)	56 (98.2)	2 (1.7)	116 (98.3)
Splinting is used as treatment in the primary dentition for						
Uncomplicated crown fracture	7 (11.5)	54 (88.5)	6 (10.5)	51 (89.5)	13 (11.0)	105 (89.0)
Complicated crown fracture	4 (6.6)	57 (93.4)	7 (12.3)	50 (87.7)	11 (9.3)	107 (90.7)
Crown‐root fracture	18 (29.5)	43 (70.5)	22 (38.6)	35 (61.4)	40 (33.9)	78 (66.1)
Alveolar bone fractures*	36 (59.0)	25 (41.0)	37 (64.9)	20 (35.1)	73 (61.9)	45 (38.1)
Root fractures*	29 (47.5)	32 (52.5)	28 (49.1)	29 (50.9)	57 (48.3)	61 (51.7)
Concussion	10 (16.4)	51 (83.6)	2 (3.5)	55 (96.5)	12 (10.2)	106 (89.8)
Subluxation	30 (49.2)	31 (50.8)	20 (35.1)	37 (64.9)	50 (42.4)	68 (57.6)
Extrusive luxation	38 (62.3)	23 (37.7)	37 (64.9)	20 (35.1)	75 (63.6)	43 (36.4)
Lateral luxation*	47 (77.0)	14 (23.0)	36 (65.6)	21 (34.4)	83 (70.3)	35 (29.7)
Intrusive luxation	24 (39.3)	37 (60.7)	28 (49.1)	29 (50.9)	52 (44.1)	66 (55.9)
Avulsion	29 (47.5)	32 (52.5)	31 (54.4)	26 (45.6)	60 (50.8)	58 (49.1)
Unsure	5 (8.2)	56 (91.8)	5 (8.8)	10 (8.47)	10 (8.5)	66 (55.9)
**Fractures and Subluxation Injuries to Permanent Teeth**						
Splinting is used as treatment in the permanent dentition for:						
Uncomplicated crown fracture	8 (13.1)	53 (86.9)	4 (7.0)	53 (93.0)	12 (10.2)	106 (89.8)
Complicated crown fracture	8 (13.1)	53 (86.9)	6 (10.5)	51 (89.5)	14 (11.9)	104 (88.1)
Alveolar bone fractures*	43 (70.5)	18 (29.5)	41 (71.9)	16 (28.1)	84 (71.2)	34 (28.8)
Root fractures*	40 (65.6)	21 (34.4)	33 (57.9)	24 (42.1)	73 (61.9)	45 (38.1)
Concussion	7 (11.5)	54 (88.5)	5 (8.8)	52 (91.2)	12 (10.2)	106 (89.8)
Subluxation	28 (45.9)	33 (54.1)	19 (33.3)	38 (66.7)	47 (39.8)	71 (60.2)
Extrusive luxation*	52 (85.2)	9 (14.8)	43 (75.4)	14 (24.6)	95 (80.5)	23 (19.5)
Lateral luxation*	52 (85.2)	9 (14.8)	42 (73.7)	15 (26.3)	94 (80.0)	24 (20.3)
Intrusive luxation*	42 (68.9)	19 (31.1)	35 (61.4)	22 (38.6)	77 (65.3)	41 (34.7)
Avulsion*	51 (83.6)	10 (16.4)	46 (80.7)	11 (19.3)	97 (82.2)	21 (17.8)
Unsure	5 (8.2)	56 (91.8)	4 (7.0)	53 (93.0)	9 (7.6)	109 (92.4)
Other	0 (0.0)	61 (100)	2 (3.5)	55 (96.5)	2 (1.7)	116 (98.3)

* Indicates correct answer.

*Knowledge and self‐reported confidence levels of dental students towards TDI management* Overall, the majority of students (70.3%) had an average level of knowledge in managing TDI, with a mean score of 26.2 ± 5.8 (Table 4). Fourth year students in 2022 had a higher mean score of 27.8 ± 5.4, compared to fourth year students in 2023 who had a mean score of 24.6 ± 5.8, resulting in a mean difference of 3.2 (*p* = 0.003).

**TABLE 4:** Distribution of knowledge levels and confidence levels.

	Year 4 (2022) *n* (%)	Year 4 (2023) *n* (%)	Total combined *n* (%)	*p* value (2022 vs. 2023)
Knowledge level				0.003
Below average (0.0%–49.9%)	5 (8.2)	18 (31.6)	23 (19.5)	
Average (50.0%–74.9%)	47 (77.0)	36 (63.2)	83 (70.3)	
Above average (75.0–100.0%)	9 (14.8)	3 (5.3)	12 (10.2)	
Confidence level				0.02
Below average (0.0%–49.9%)	52 (85.2)	47 (82.5)	99 (83.9)	
Average (50.0%–74.9%)	9 (14.8)	8 (14.0)	17 (14.4)	
Above average (75.0–100.0%)	0 (0.0)	2 (3.5)	2 (1.7)	

A high percentage of participants (83.9%) reported low confidence in managing trauma cases. Mean knowledge scores were also tabulated for each confidence level category. A significant relationship was found between knowledge and confidence levels (*p* = 0.013) (Table 4). Overall, the independent *t*‐test showed a significant difference between confidence scores in fourth year 2022 and 2023 students (*p* = 0.02).

## 4 Discussion

This study offers insight into the TDI management knowledge amongst dental students across the 2022 and 2023 cohorts. These students had an average level of knowledge in managing TDI (59.6% correct answers). This is consistent with studies from Japan, India, and the United Kingdom which reported moderate levels (50% to < 70% correct answers) of TDI management knowledge amongst their dental students [14, 17, 19]. Although this may seem acceptable, 59.6% sits on the lower range of the average score and is consistent with the systematic review by Tewari et al. *(2021)* which found that knowledge of TDI management amongst dental students was insufficient [17].

The most significant gap in TDI knowledge was in the management of avulsed permanent teeth, followed by fractures and subluxations in permanent teeth and lastly, the management of primary teeth. Avulsion is one of the most serious TDI, with a prevalence of 16% in both primary and permanent dentitions [20]. The prognosis of a replanted avulsed tooth is dependent on several factors, including extra‐oral period, endodontic treatment post‐replantation, splinting and storage media, which were assessed in this study. In cases of avulsion of a mature tooth, endodontic treatment is crucial to prevent or stop the progression of complications [21]. Only 12.7% of the participants in the current study would commence endodontic treatment within 2 weeks after replanting a mature avulsed tooth. Several research and review papers have recommended that root canal treatment should be commenced immediately [22] following replantation and stabilisation of an avulsed tooth or within 7–14 days [11] when pulp revascularisation cannot be expected [5, 20, 22]. When root canal treatment was commenced after 3 weeks, a higher frequency of external inflammatory resorption was reported [23]. Splinting stabilises, protects and immobilises an avulsed tooth in the socket, while promoting pulp and periodontal healing [24, 25]. Long‐term and rigid splinting may cause ankylosis and replacement resorption [26]. In the current study, 18.6% of the students understood the splinting protocols of 2 weeks for an avulsed tooth [11]. This is significantly lower than in other studies [24, 25, 26].

**FIGURE 1:**
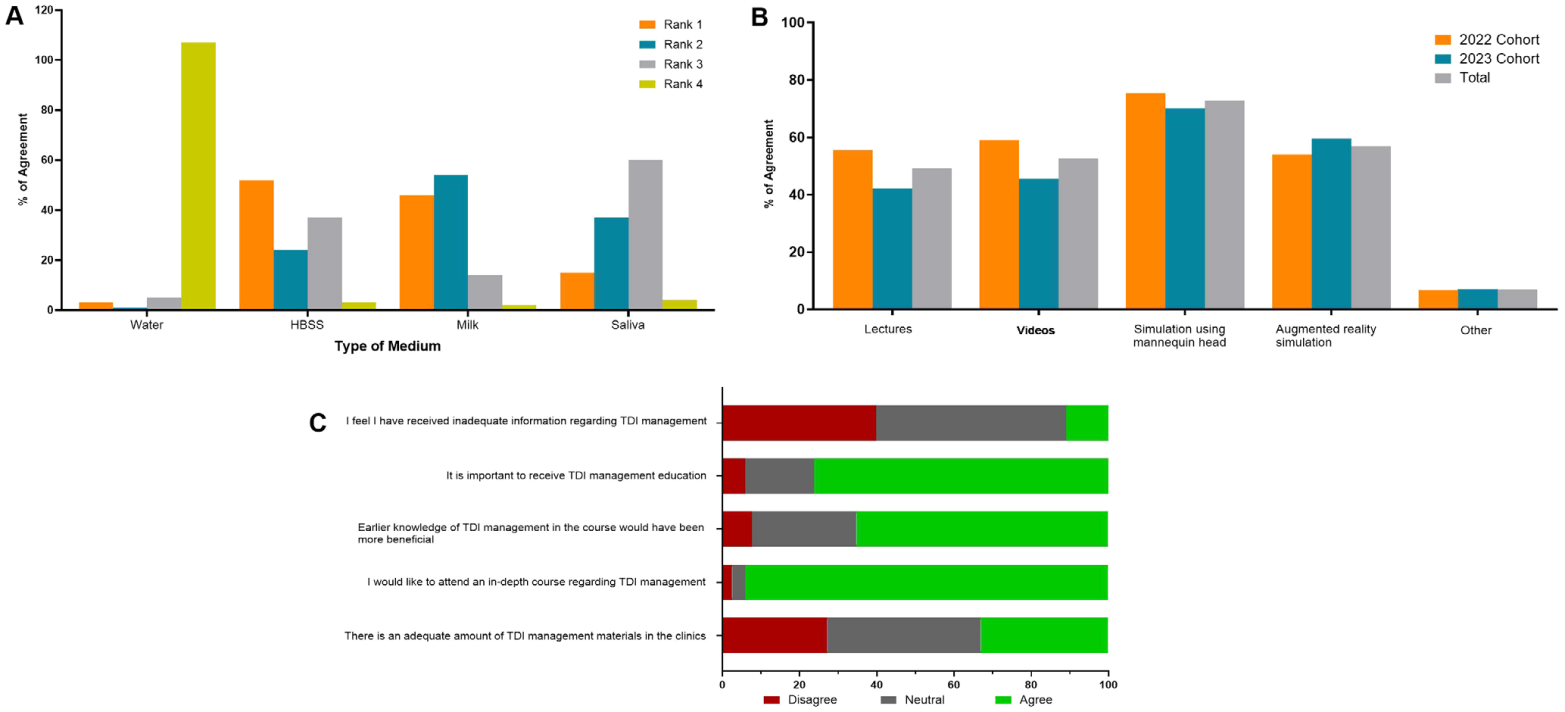
Distribution of responses collected for (A) Participants' ranking of storage medium for an avulsed tooth (B) Participants' preference of learning media (C) Participants' attitudes towards TDI management.

The type of storage media in which the avulsed tooth is placed also affects its prognosis as it influences the viability of the PDL cells; therefore, potentially leading to ankylosis and external replacement resorption, and it may also affect pulp healing [27]. In the current study, only 39.0% of the students correctly identified milk as their first choice of storage media for an avulsed tooth (Figure 1A). This is lower than in the studies reported by Al‐Shamiri et al. [13] and Fujita et al. [14] who showed that 77.0% and 57.4%, respectively, of dental students in their studies chose milk [13, 14]. Milk has an optimal pH of 6.5–7.2 and a combination of nutrients that allows it to keep PDL cells viable [28]. Additionally, milk is commonly available and it is cost effective, which makes it a very practical storage medium [28]. Saliva is also a very practical and effective storage medium since it will always be available at the accident site, although the PDL cells may not survive beyond about 60 min. Water is the least desirable storage medium for an avulsed tooth as the hypotonic environment causes rapid cell lysis and inflammation on replantation [29]. HBSS is an isotonic solution and can maintain periodontal ligament cell viability for extended periods but it is often not available at accident sites and hence, it can be deemed as an impractical storage medium [29]. The above reasons and the response of the students in the current study suggest that they were deficient in their knowledge of the appropriate management of avulsed permanent teeth.

Appropriate urgent care is essential to ensure a good prognosis following trauma to a tooth. Therefore, it is important to have knowledge of the diagnosis and immediate management protocols for all the various TDI as recommended in the IADT guidelines [9, 10, 11, 12]. The revised 2020 IADT guidelines provide easily accessible information on TDI management, supported by evidence‐based literature and dental trauma experts [9, 10, 11, 12]. The current study has shown that the mean knowledge score for those students who had heard of the IADT guidelines was 27.6 ± 4.8, while those who had not heard of the guidelines had a significantly lower mean score of 18.2 ± 6.0 (Table 1). The mean knowledge score for those students who had read the IADT guidelines was 28.1 ± 4.6, while those who had not read the guidelines had a lower mean knowledge score of 26.2 ± 6.0 (Table 1). There was a significant correlation between knowledge score and those who had heard of (*p* = 0.003) and had read the IADT guidelines (*p* = 0.003) (Table 1). This is corroborated by Mazur et al. who concluded that cognizance of the IADT guidelines amongst general dentist practitioners resulted in better overall knowledge scores [30]. This highlights the significance of understanding the IADT guidelines to achieve successful TDI outcomes for both dentists and dental students.

The present findings indicate a high percentage of students (83.9%) had low confidence, with a below average mean confidence level of 6.93 ± 4.3 (Table 4). Similarly, AlZoubi et al. reported a low mean confidence level of 2.41/10 among dental students [19]. In the present study, a significant relationship between knowledge and confidence level was noted (*p* = 0.013). In contrast, several studies found that self‐evaluated confidence was not an accurate reflection of actual knowledge level in handling TDIs [31, 32]. Rather, the authors postulated that recency of education or attendance at continuing education courses may have a greater impact on self‐confidence than clinical experience [31]. Confidence levels have been shown to increase after education has been provided, suggesting the importance of continuous education for dental trauma [17]. Therefore, exploring creative learning modes such as virtual reality, simulation clinics and other clinical components can help to advance knowledge and confidence levels in TDI management (Figure 1B). The current undergraduate dental traumatology module has strengthened the students' attitude towards managing TDI injuries, with 94.0% of participants in agreement or strong agreement of the importance of the TDI module being taught as part of the university dental curriculum (Figure 1C) and 76.3% of the participants showed interest in attending further modules to improve their current TDI knowledge (Figure 1C).

To the best of the authors' knowledge, this is the first study to investigate Australian dental students' knowledge of TDI management. Assessing current TDI knowledge has revealed areas of inadequacy in the dental students' TDI knowledge and where the current dental traumatology module could be improved. This study is limited in its cross‐sectional nature. A significant difference in knowledge between the 2022 and 2023 cohorts (*p* = 0.003) was observed in this study, with the 2022 cohort faring better than the 2023 cohort across all areas of TDI management (Table 4). While the two cohorts of participants were in their fourth year of studies at the time of completing the questionnaire, plus they had experienced the same TDI module and curriculum, intrinsic abilities in comprehending TDI could have resulted in the differences not accounted for. Moving forward, a longitudinal study which follows the same cohort of students could be beneficial in determining how the TDI module impacts different years of their studies as well as after they graduate as dentists. The focus on a small group of participants, particularly the fourth‐year dental students, is another limitation of this study. The small sample size is attributed to this study being conducted in the aftermath of the COVID‐19 pandemic where travel restrictions made it difficult to hold the yearly Australian dental student conference in which dental students from all across Australia could come together to attend. Therefore, this made it difficult to disseminate the TDI questionnaire to a large audience. Future studies with larger sample sizes including dental students from other Australian universities are recommended to draw more conclusive results. Additionally, the inclusion of fifth year dental students in future studies could also identify how clinical experience affects dental students' TDI knowledge, as the fifth‐year students would be exposed to more clinical cases while on their paediatric rotations in their final year placements. In support of this, Jadav and Abbott (2022) showed a proportional increase in knowledge score with increased clinical experience in registered Australian dentists [1]. Further investigations could be done to identify if there is a similar trend among Australian dental students. However, this study provides crucial preliminary information on how to better facilitate the delivery of the TDI module which is fundamental in training dental students to manage TDI cases.

## 5 Conclusions

This study demonstrated that dental students' knowledge of dental trauma management based on the IADT guidelines is average, with deficiencies in managing permanent tooth avulsion. In general, students were also lacking confidence in managing TDI cases. The study also highlights that dental students have limited clinical exposure to dental trauma cases in dental school, suggesting the need for more innovative learning methods such as case‐based learning and interactive augmented reality simulations as a substitute for direct clinical contact.

## Author Contributions

S.Z. and P.A. conceived the idea. L.L.J.C., V.L. and A.M.T. collected the data, analysed the data and led the writing. S.Z. and P.A. revised the manuscript and gave final approval of the version to be published and agreed to be accountable for all aspects of the study.

## Conflicts of Interest

The authors declare no conflicts of interest.

## Data Availability

The data that support the findings of this study are available on request from the corresponding author. The data are not publicly available due to privacy or ethical restrictions.
